# Vulnerability of Southern Afar pastoralists to climate variability and change, Ethiopia

**DOI:** 10.4102/jamba.v11i1.575

**Published:** 2019-04-18

**Authors:** Muluken Fenta, Andries Jordaan, Yoseph Melka

**Affiliations:** 1 Disaster Management Training and Education Centre for Africa, University of the Free State, Bloemfontein, South Africa; 2 Wondo Genet College of Forestry and Natural Resources, Hawassa University, Shashemene, Ethiopia

## Abstract

The present study was carried out in the Southern Afar region in Ethiopia to assess the vulnerability of pastoral communities to climate change and variability. A household questionnaire survey was employed to collect data at a household level. A total of 250 pastoral households were sampled using stratified random sampling. The results revealed that 28.8% of the pastoral households were highly vulnerable. Most of the households (53.6%) were moderately vulnerable. Only 17.6% of the households were capable of coping even though there would be a high probability of moving from less vulnerable to a moderate or high vulnerability level in the future if no appropriate adaptive measures would be taken by decision-makers. Policies with emphasis on empowerment of women, such as improving their access to and control over resources through a better institutional set-up; improving irrigation facilities and skills; expanding the participation of pastoral households on irrigation farming; creating opportunities for non-farm income; and improving access to credits, markets, health and veterinary services, are expected to enhance pastoralists’ resilience.

**Keywords:** climate; irrigation; livestock; pastoralists; vulnerability.

## Introduction

In pastoral communities of Ethiopia, climate-induced shocks and stresses, such as droughts, rising temperature and irregular rainfall, reduced the extent of pastoral areas and negatively impacted water availability and led to animal deaths owing to hunger and diseases (Conway 2000). The weather-related natural disasters frequently occurred in pastoral areas of Ethiopia, which has been further exacerbated by the depletion of the natural resources and destruction of ecosystems because of anthropogenic activities such as deforestation and allocation of grazing areas to commercial farms (Tadege 2007). Ethiopia is particularly very susceptible to drought, which is the most significant climate-change-related disaster influencing the country over time (Seleshi & Zanke 2004). Rainfall anomalies and the delayed onset of the rainy season, along with rising temperatures, lead to impoverishment of grassland, lack of livestock feed and water as well as heat stress to livestock. This has, in turn, increased the mortality rate of herds, susceptibility of livestock to disease and emaciation as a result of the long distances they travel in search of pasture and water (Muluneh & Demeke 2011). Droughts, floods and heat waves have increased in Ethiopia over the past decades. Drought will affect all nations, but the impact will be higher on low-income countries, such as Ethiopia, which have limited capacity to cope with the effects of the same (Funk et al. 2008; Seleshi &Zanke 2004; Williams & Funk 2011). Drought continues to be a major challenge for the Ethiopian people (United Nations 2008), and in the 21st century, there has been an increasing frequency of extreme droughts because of global warming in Ethiopia (Institute of Development Studies 2008). The country has confronted severe droughts at least twice per decade for the last five to seven decades, with the most serious ones in 1972–1973, 1984 and 2002–2003 (Mideksa 2010, Tadege 2007). Besides, flood is also a climate-related disaster that happens in Ethiopia from time to time. In 2006, flood caused significant loss of property and human life in many regions of Ethiopia (Tadege 2007). Moreover, regional projections of climate models predicted a rising frequency of extreme flooding because of global warming in Ethiopia (Institute of Development Studies 2008). Over the past 55 years, there has been a warming trend also in Ethiopia. According to Tadege (2007), the temperature has been increasing by about 0.37 °C every 10 years. This increase in temperature consequently gives several impacts and effects such as heat stress to livestock, and high evapotranspiration.

It has been observed that although climate change is a world-wide phenomenon, its influence and extent differ across multiple levels and scales. Its impacts are not the same at district, regional, national and global levels. Although changes in climate and climate extremes will be the greatest challenge for people in Ethiopia, few studies have been undertaken in the country on vulnerability to climate change. Most of this literature has only investigated poverty and food insecurity (Dercon & Krishnan 2000). Deressa, Hassan & Ringler. (2008) assessed the vulnerability of households to climate-induced shocks and stresses at the national level in Ethiopia. However, insights into vulnerability to climate perpetuation vary with the scale of analysis. Vulnerability to climate-induced shocks and stresses assessed at the national level can conceal variations in vulnerability of households at the local level (Parkins & MacKendrick 2007). Accordingly, this national-level (macro-scale) assessment by Deressa et al. (2008) could have overlooked variations in vulnerability at the local level because the vulnerability level may vary even among households at the district level. Households at the district level can vary in terms of the level of food insecurity, coping and adaptation capacity, access to credits, public services, safety nets and natural resources. In such conditions, variability at the local level is usually ignored in nationwide vulnerability studies. Therefore, it is difficult to precisely understand the spatial aspects of households’ vulnerability from nationwide vulnerability assessments. This shows the significance of scale in vulnerability studies and ensures the necessity of vulnerability study at the micro level. It is on the basis of these premises that the present study needs to understand the vulnerability of pastoralists to climate change and variability in the Southern Afar region of Ethiopia.

## Methodology

### Study areas

**FIGURE 1:**
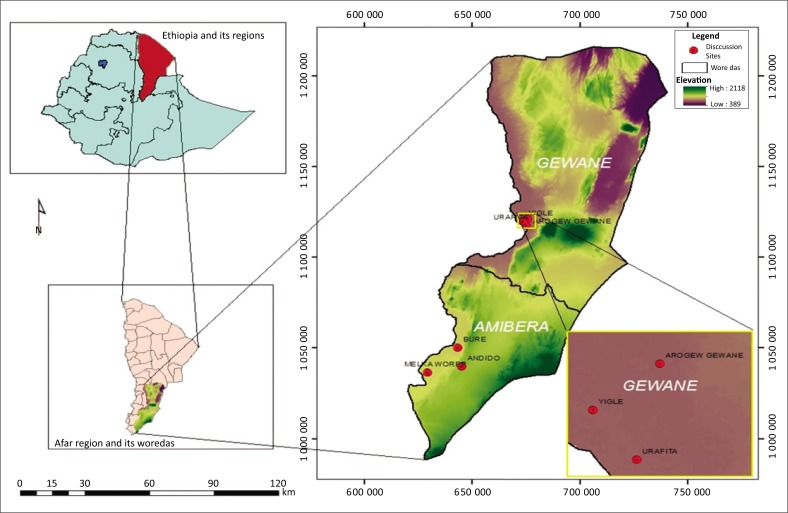
Map of the study area.

The Afar region is situated in the northeastern part of Ethiopia and comprises about 270 000 km^2^ (CSA 2008) between 39°34’E and 42°28’E and 8°49’n and 14°30’N. The study was conducted in the Southern Afar region in Amibara and Gewane districts (see Figure 1). Agro-ecologically, the climate of Amibara is generally semi-arid with a temperature level that falls between 25 °C and 35 °C and an average annual rainfall below 600 mm. The altitude of Amibara ranges from 720 m.a.s.l. to 1100 m.a.s.l. The altitude of Gewane ranges from 550 m.a.s.l. to 650 m.a.s.l. and its temperature falls between 28 °C and 42 °C, with an average annual rainfall below 500 mm (CSA 2008).

### Research design

A stratified random sampling approach was adopted to sample villages and households. Stratification was based on livelihood activities practiced by households in the two districts, Amibara and Gewane. Firstly, villages were identified and stratified into pastoralists (those households are solely dependent on livestock production) and agro-pastoralists (those households are dependent on both livestock and crop production). Accordingly, from the Amibara district comprising 10 pastoralist and five agro-pastoralist villages, three villages consisting of two pastoralist villages (Andido and Bure) and one agro-pastoralist village (Melka-Worer) were randomly selected using the lottery system. Similarly, from the Gewane district, which involves six agro-pastoralist and two pastoralist villages, three villages consisting of two agro-pastoralist villages (Yigle and Urafita) and one pastoralist village (Arogew Gewane) were randomly selected. Overall, six villages were sampled from the two districts.

After random selection of the study villages, the total number of households was obtained from the district pastoral and agricultural development officer. A formula by Krejcie and Morgan (1970) was followed to determine the sample size in each of the villages sampled. For the selected households whose heads were absent, the next household was chosen and interviewed; a total of 250 households were interviewed from Amibara and Gewane districts.

An open- and closed-ended questionnaire was administered face to face with the heads of the households within six villages of the Amibara and Gewane districts from December 2016 to February 2016. The collected data were coded and thereafter analysed using Statistical Packages for the Social Sciences (SPSS) (version 20). To complement the household questionnaire data, 29 individuals from sampled villages and various organisations were interviewed as key informants. Four focus group discussions were selected separately with gender equality (six men and six women) from the sampled villages.

### Data collection

A pilot test run was undertaken with local enumerators and key informants before the beginning of the household interviews, and the final questionnaires were revised and rewritten accordingly. The questionnaire used for the pre-test was excluded from the final data entry and analysis. The piloting was carried out to check the suitability of the tools and also whether the field assistants could manage the questionnaire without difficulty.

Information on various aspects was collected through interviewing of the selected household head. The survey addressed information about household characteristics, household access to basic services, livelihood assets and their trends, income per household, sources of income, climate change information, climate impacts, adaptation and coping strategies, farm labour, social networks and remittances. To avoid misunderstanding, the household interviews were undertaken in the local language by the local field assistants.

### Assessing vulnerability of pastoralists to climate variability and change

This study used vulnerability indicators to assess vulnerability of pastoralists to climate shocks and stresses. The method for selection of vulnerability indicators is discussed as follows: Vulnerability to perturbation involves different dimensions and is influenced by interrelated multiple components. Most indicators of vulnerability are latent variables, and hence, difficult to measure. However, based on vulnerability indices, the vulnerability levels of households can be assessed. To develop indices, the appropriate indicators of social, economic and environmental vulnerability must first be selected before weights can be assigned for each indicator. Lastly, these indicators are combined to develop an index. Indices are valuable to represent a complex reality in simpler terms. However, the approach employed to select indicators is very significant, as the selection of non-representative indicators can result in the development of the wrong index of vulnerability. Theory-driven and data-driven are the two approaches in the choice of indicators (Vincent 2004). The choice of appropriate indicators can be carried out depending on certain theories that offer understanding of the determinants of vulnerability. Nevertheless, even theory-based methods are influenced by data restrictions and subjectivity is mostly a problem during the selection of indicators. The appropriate alternative is to validate the representativeness of the theory-based indicators based on focus group discussions with key informants. The present study employed this approach while choosing the indicators to determine vulnerability of households in the study area.

Having selected the appropriate vulnerability indicators, the values of the vulnerability indicators were normalised to make the indicator’s value within a similar range (Gbetibouo, Ringler & Hassan 2010; Nelson et al. 2010; Vincent 2004). Normalisation is performed by subtracting the mean from the observed value and dividing by the standard deviation for each indicator.

$$Normalised\;value=\frac{Observed\;Value-Mean}{Standard\;deviation}.$$ [Eqn 1]

The next step was to assign weights to the selected vulnerability indicators. In this study, the principal component analysis (PCA) was employed to assign weights to the indicators (Filmer & Pritchett 2001). The normalised variables were then multiplied by the weights of the corresponding indicators, using the following formula:

$$I_{j}=\sum_{i=1}^{K}b_{i}\left(\frac{a_{ji-X_{i}}}{S_{i}}\right)$$ [Eqn 12]

where:

*I* = the index value

*b* = the weights from PCA

*a* = the individual value of the indicator

*x* = the mean value of the indicator

*s* = the standard deviation of the indicators.

Lastly, the vulnerability index of each household was calculated using the following equation (following IPCC 2012):

V=AC−(E+S), [Eqn 3]

where:

*V* = the vulnerability index of each household

*AC* = the adaptive capacity index

*E* = the exposure index

*S* = the sensitivity index for the corresponding household.

The model specification is further described as follows:

$$\begin{array}{l} V_{i}=\left(A_{1}X_{1j}+A_{2}X_{2j}+\ldots +A_{n}X_{nj}\right)-\\ \left(A_{n+1}Y_{1j}+A_{n+2}Y_{2j}+\ldots A_{n+n}Y_{nj}\right), \end{array}$$ [Eqn 4]

where:

*V_i_* = the vulnerability index

*X_s_* = elements of adaptive capacity

*Y_s_* = elements of sensitivity and exposure

*A_s_* = the factor score of each variable computed using PCA.

The values of *X* and *Y* are obtained by normalisation using their mean and standard errors (see Eqn 4). This study used an integrated approach, which combined social, economic and environmental indicators to construct vulnerability indices for each household as suggested by Madu (2012) and employed by Tesso, Emana and Ketema (2012) in Ethiopia. The vulnerability index was suggested after the definition given by the IPCC (2012) that vulnerability is considered as a function of adaptive capacity, sensitivity and exposure of the system.

When the sensitivity and exposure of the households are lower than that of its adaptive capacity, the household becomes less vulnerable to climate-induced hazards and vice-versa. The assessment of the socio-economic vulnerability approach gives emphasis to the socio-economic and political conditions of the local people. Vulnerability levels among people in a given area can differ according to their level of knowledge and skills, income level, welfare, access to affordable credits and inputs such as improved varieties, access to early warning information, social capital and political ability (Füssel 2007). The biophysical approach measures the degree of harm because of climate perturbations and stresses on the systems (Jones et al. 2004). The biophysical, or impact assessment, approach is mostly focused on the physical influence of change in climate on various features of the system.

## Results and discussions

### Descriptive statistics

The results indicated that the average size of the family in the study area consists of about eight people. This was relatively higher than the national average rural household size. Such large family size in the region might be associated with the polygamy culture that is commonly practiced in the Afar region. The average age of household heads was 52.7 years and most of them were men (58.8%). The majority of respondents (72.2%) were found to be illiterate, which means that only 28.8% of the respondents could read and write, with a formal education ranging from 1 to 10 years. Furthermore, the results revealed that only 28.8% of the households had access to extension services, which suggests that access to extension services in the Southern Afar region was very poor.

On the other hand, access to basic services in the Southern Afar region was generally poor. For example, only 22.2% of the households had access to markets near their villages. The majority of the households (77.8%) usually travel long distances to sell their cattle and camels. According to the respondents, on average, they travel for more than 12 h across other adjacent districts or regions (Amhara and Oromia) to get to the market for selling large animals (cattle and camels), which is costly in terms of time and labour. During such long-distance travelling, pastoralists sometimes lose their livestock because of stresses associated with long travelling, feed and water scarcity and motor vehicle accidents. The animals that finally make it to the market would have lost weight, which in turn has an adverse effect on the selling price. Sheep and goats are sold in a nearby small village market but because of a lack of bargaining power and not enough purchasers, they cannot sell their livestock for a reasonable price. The results further showed that only 30% of the households had access to veterinary clinics and services and 32% of households had access to the health centre and health services.

The results further indicated that households’ access to credit was very poor in the study area. Only 14.8% of households had access to credit. Furthermore, 90% of the households received market information about the price prior to selling through an informal local information exchange system (locally called *Dagu*). Only 2% of the households received formal market information through district extension officers and the radio. Therefore, there is a need to provide affordable credit access and market information in order to enhance the resilience of pastoralists towards climate-induced shocks and stresses.

### Hazards perceived by local people

According to the local respondents, the rainfall of late has become more unpredictable and erratic, this rainfall variability turns into frequent and prolonged droughts. The findings indicated that drought was the most frequent hazard in the study areas as reported all the respondents (*n* = 250), followed by the encroachment of the rangeland by invasive species, mostly *Prosopis juliflora*, livestock diseases, loss of dry season grazing areas and floods (see Table 1).

**TABLE 1:** Hazards perceived by sample households (multiple responses were possible).

Perceived hazards	Number of respondents (*N* = 250)	Percentage
Droughts	250	100.0
Rangeland encroachment by invasive species	230	92.0
Livestock diseases	213	85.2
Loss of dry season grazing areas	195	78.0
Floods	105	42.0

The results showed that cattle diseases such as blackleg, locally referred to as *harayti*, and trypanosomiasis, locally called *sole*, and diseases of camels such as *geramole* and *gosso* were some of the frequent hazards threatening their cattle and camels. Contagious caprine pleuro-pneumonia, a fatal disease that mainly affects goats, and mange, a skin disease caused by parasitic mites, were also the most common diseases affecting sheep and goats in the study areas. Respondents further complained that allocation of the dry season grazing areas for commercial farmers was also another hazard curtailing livestock mobility during dry seasons, increasing vulnerability of pastoralists to climate change and variability.

### Vulnerability indicators

The socio-economic and biophysical vulnerability indicators selected for this study are indicated in Table 2. The results showed that 76.8% of the households had more than five family members, with 32% respondents reporting that they had more than three dependents, aged less than 15 years and 65+ years. Pastoralism is a livelihood strategy requiring more labour availability. Thus, those households with more dependents, aged less than 15 years and 65+ years, were more vulnerable to climate-related hazards. The findings further showed that 72.2% of the households were illiterate. This, in turn, decreases households’ capability to access markets, climate and early warning information, making households more vulnerable to climate-induced shocks and stresses.

**TABLE 2:** Selected vulnerability indicators and their effects on vulnerability.

Hypothesised vulnerability indicators	Percentage	Effects on vulnerability
Gender of household head: female-headed	41.2	+
Age of household head: 50+ years	46.8	+
Marital status: single	30.6	+
Household size: more than 5 persons	76.8	−
Dependents: >3 persons	32	+
Educational level: unable to read and write	72.2	+
Member of household sick or died associated with climate-related hazards	30.4	+
Extension services: having no extension services	72.2	+
Linkages: having social linkages	63.2	−
Distance to health service: more than 10 km	68.0	+
Access to EWI: no access to information	82.0	+
Experience: >5 years of farming experience	3.6	−
Livestock owned: having less than 2 TLU	26.8	+
Irrigation farming: having practiced irrigation farming	40.0	−
Non-farm income: have non-farm income sources	60.0	−
Mobility: ability to move freely	57.6	−
Radio owned: having a radio	18.2	−
Access to remittances: having cash transfer	30.0	−
Distance to market: more than 10 km	77.8	+
Distance to veterinary clinic: more than 10 km	70.0	+
Credit access: no credit access	85.2	+
Access to agricultural inputs	24.4	−
Households having food shortages during normal season of the year	60.6	+
Rainfall: experience decrease rainfall	98.0	+
Temperature: experience increasing	95.0	+
Households facing flood hazards two or more times in 10 years	42.0	+
Households experiencing increasing frequency of droughts	100.0	+

TLU, tropical livestock unit (1 TLU is equivalent to 250 kg); EWI, early warning information.

+, Positive sign shows that the variable increases vulnerability; −, negative sign shows that the variable decreases vulnerability.

As shown in Table 2, more than 41% of the household heads were women who were more vulnerable to climate-induced shock and stresses as they had low access to assets, credits, social participation and climate information. On the other hand, 72.2% of the households had no access to extension services, indicating their vulnerability to climate-induced shocks such as droughts. The results imply that the education level of the head of the household, and access to extension services and climate information were the most important social vulnerability variables in the study area. The findings further indicated that approximately 60% of the households had no multiple livelihood opportunities and most of them were dependent on livestock production for their incomes (see Table 2). Forty per cent of the households practiced irrigation crop farming alongside livestock keeping. The results also revealed that 77.8% and 70% of the households complained that they had to travel more than 10 km to access markets and veterinary services, respectively.

Moreover, the findings showed that about 57.6% of the households practiced livestock mobility as a coping strategy against drought in the study area (see Table 2). In addition, 85.2% of the respondents noted that they had no credit services in the study area. The results further revealed exposure to climate shocks and stresses (see Table 2). Ninety-eight per cent of the respondents indicated that they experienced decreasing rainfall, while 95% of the households experienced increasing temperature for the last three decades. Furthermore, all households experienced increasing frequency of droughts, while 42% of the households faced flood hazards two or more times in the last 10 years.

### Measuring vulnerability of households

In the present study, PCA was conducted to develop the vulnerability indices and measure the vulnerability of households quantitatively. Twenty-seven indicators of household vulnerability were studied and their factor scores are indicated in Table 4. The appropriateness of the data was assessed depending on the Kaiser-Meyer-Olkin (KMO) and Bartlett’s tests values before running the factor analysis. According to Li and Weng (2007), if the KMO value is greater than 0.5 and the Bartlett’s test value is less than 0.1, the factor analysis can be run. It is observed that the KMO measure of sampling adequacy was 0.728, indicating that the model was fairly acceptable (Table 3).

**TABLE 3:** Kaiser–Meyer–Olkin and Bartlett’s test.

Test	Result
Kaiser–Meyer–Olkin	
Measure of sampling adequacy	0.728
Bartlett’s Test of Sphericity	
Approximate Chi-Square	1487.120
Df	210
Sig.	0.001

Df, Degree of Freedom; Sig., significance.

Having checked the appropriateness of the data for PCA analysis, PCA analysis was carried out on the vulnerability indicators listed in Table 4. The results of PCA revealed that three components were extracted with eigenvalues greater than 1 explaining 77.36% of the total variation. It is observed that 52.33%, 14.29% and 10.74% of the variation were explained by the first, second and third principle components, respectively. The factor scores and their relationship with the vulnerability indicators are indicated in Table 4. The household’s vulnerability index was developed using Equation 4, explained in the section titled ‘Assessing vulnerability of pastoralists to climate variability and change’. Based on the developed vulnerability index, households were grouped into three vulnerability levels. Those households having a vulnerability index of 3.0–5.9 were grouped as less vulnerable and accounted for 17.6% of the households (see Table 5). Less vulnerable households are in a vulnerable state, but can still cope. Similarly, households grouped as moderately vulnerable are those that need immediate, but short-term, support during climate-induced shock and had a vulnerability index of -2.5 to +2.9 and constituted 53.6% of the sampled households. The third vulnerability group comprised highly vulnerable households having a vulnerability index of -2.51 to -4.49. Highly vulnerable households are those that are at the emergency level, and they accounted for 28.8% of the sampled households (see Table 5).

**TABLE 4:** Factor loadings of vulnerability indicators from principal component analysis.

Vulnerability indicators	Factor score
Gender of household head	0.637
Age of household head	−0.762
Marital status	0.537
Household size	0.827
Dependant ratio	−0.504
Educational level	0.780
Member of household sick or died associated with climate-related hazards	0.542
Extension services	0.874
Social linkages	0.702
Distance to health service	0.574
Access to EWI	0.604
Farming experience	0.501
Livestock owned	0.967
Irrigation farming	−0.807
Non-farm income	0.664
Livestock mobility	0.731
Radio owned	−0.594
Access to remittances	0.569
Distance to market	0.789
Distance to veterinary clinic	0.735
Access to credits	0.813
Access to agricultural inputs	−0.819
Households having food shortage during normal season of the year	0.578
Experiencing decrease rainfall	−0.956
Experiencing increasing temperature	−0.924
Households facing flood hazards	−0.587
Households facing drought hazards	−0.834

EWI, Early Warning Information.

**TABLE 5:** Vulnerability levels and situations of pastoral households.

Levels of vulnerability	Situation of households	Vulnerability index	Percentage of households
Highly vulnerable	The most susceptible households even for slight shock and need intensive care	−2.51 to −4.49	28.80
Moderately vulnerable	Households who need temporary support to recover when they are hit by hard climate-induced shock	−2.50 to +2.59	53.60
Less vulnerable	Coping households – household in a susceptible situation but still capable to cope	+3.00 to 5.90	17.60

Total	-	-	100.00

**FIGURE 2:**
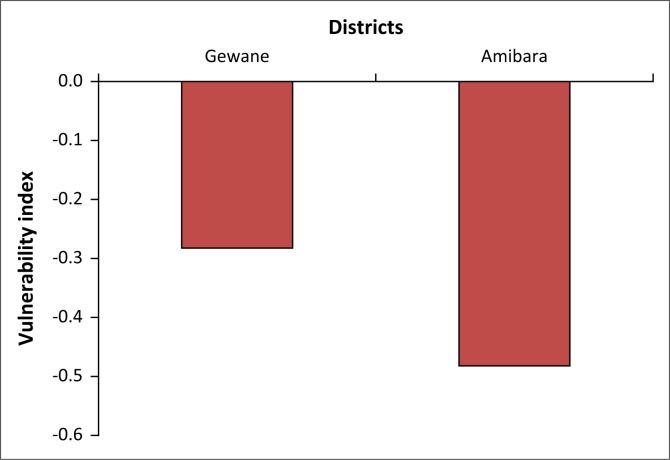
Vulnerability levels of households by district.

Furthermore, this study compared vulnerability of households by livelihood groups, districts and gender of the household head. Accordingly, the findings indicated that households living in the Gewane district were relatively less vulnerable than households living in the Amibara district, although significant difference was not observed between the vulnerability indexes of the two districts (see Table 6 & Figure 2). This can be explained by differences in exposure to climate shocks and stresses between the two districts. The local people’s reports during the field survey indicated that, recently, the Amibara district was more drought-prone than the Gewane district.

**TABLE 6:** Paired sample tests for household vulnerability index clustered by livelihood strategy, districts and gender of the household head.

Variable	Paired differences			*T*	Sig. (two-tailed)
	Mean	Standard deviation	Standard error mean		
Pair 1 – VIPH–VIAPH	−3.593	3.114	0.3099216	−11.595	0.000
Pair 2 – Gewane–Amibara district	0.418	3.797	0.3836503	1.091	0.278
Pair 3 – Female- and Male-headed households	−1.249	3.469	0.3402433	−3.671	0.000

VIPH, vulnerability index of pastoral households; VIAPH, vulnerability index of agro-pastoral households; Sig, significance.

**FIGURE 3:**
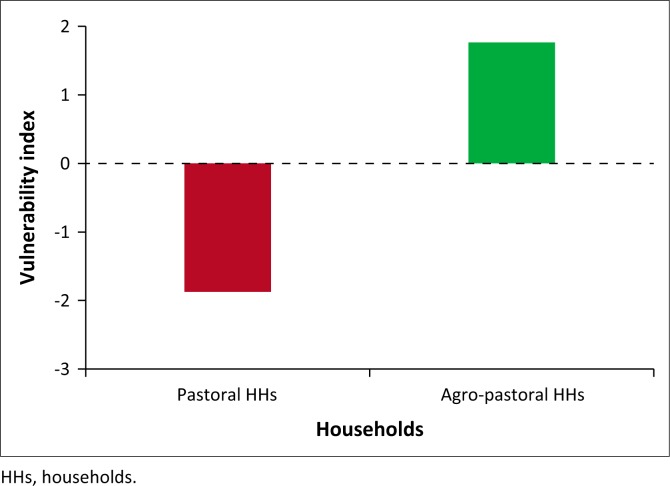
Vulnerability categories of households by livelihood strategy.

The results further indicated that agro-pastoral households were significantly less vulnerable than pastoral households (see Table 6 & Figure 3). This can be related to variations between the two groups with regard to livelihood diversification. Agro-pastoral households were practicing irrigation farming alongside livestock keeping, while pastoralists were solely dependent on their livestock as a source of income. Hence, agro-pastoral households had better adaptive capacity and were less vulnerable than pastoral households. This implies that pastoralism is becoming a risky enterprise in the Southern Afar region in a changing climate. Similar results were reported by O’Brien et al. (2004) who indicated that those households practicing irrigation farming were more adaptable to climate change and variability.

**FIGURE 4:**
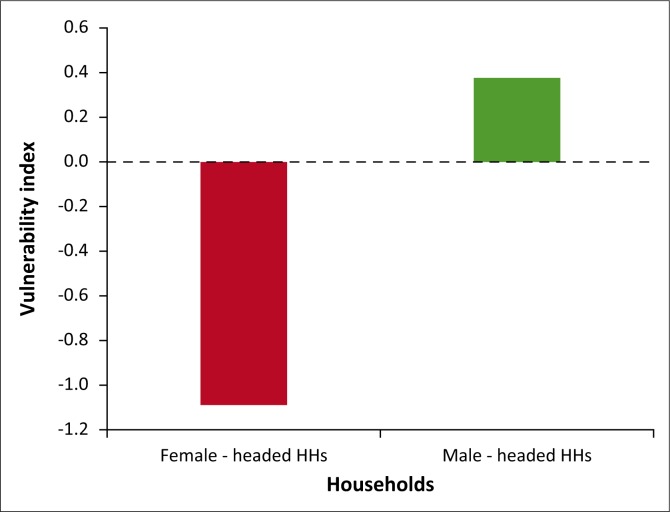
Vulnerability of households by gender. HHs, households.

The results further indicated that female-headed households were more vulnerable than male-headed households (see Table 6 & Figure 4). This was because of low adaptive capacity of women as a result of poor access to social participation, credits and assets, and their inability to combine crop farming with livestock production because of limited labour availability and low skills with regard to crop farming activities as compared to men. The results of this study are supported by Ongoro and Ogara (2012) who did their studies on the vulnerability of Samburu pastoralists in Kenya and indicated that women were more vulnerable to the impacts of climate change than their male counterparts.

## Conclusion and recommendation

Understanding the vulnerability of households to climate change and variability is indispensable for decision-makers to device adaptation strategies for long-term resilience of pastoral households. This study identified which category of livelihood groups, district and gender was more vulnerable, as this would enable decision-makers to prioritise the group of households, livelihood groups and district with regard to development intervention. Accordingly, the results revealed that 28.8% of the pastoral households were highly vulnerable. These groups of households would need intensive care and follow-up to disengage them from this situation. Most of the households (53.6%) were moderately vulnerable, suggesting that in the case of climate-induced shock, they would need some support to recover. Only 17.6% of the households were capable of coping in spite of a high probability of moving from a less vulnerable to a moderate or high vulnerability level in the future, if no appropriate adaptive measures would be taken by the decision-makers.

This study concluded that those households who practiced irrigation farming were less vulnerable than those households solely dependent on livestock production. Therefore, the Ethiopian Ministry of Federal and Pastoralist Development Affairs should enhance households’ capacity in terms of finance through provision of affordable credit access, and provide training to enhance their technical skills on crop farming. To enhance the contribution of irrigation crop farming for increasing household resilience, households should be provided with improved agricultural technologies such as a water pump for irrigation, and improved seed varieties with short growing periods and resistance to diseases. In the Southern Afar region, it was identified that lack of access to nearby markets increases vulnerability of pastoral households to climate change and variability. Therefore, interventions should be introduced to improve access to markets if pastoralists need to be resilient. Pastoralists can get the best value for their products and become more resilient if the Ministry of Federal and Pastoralist Development Affairs improves market access and develops marketing opportunities for pastoral households through establishment of the nearby market centres, road access and provision of water access along stock routes. The present study also showed that lack of access to formal early warning information increases the vulnerability of pastoralists. Therefore, early warning and early response (interventions) should be regarded as a matter of great concern by the Ministry of Federal and Pastoralist Development Affairs and should be included in the pastoral development policy for enhancing resilience of pastoralists to climate-related disasters. Furthermore, policies with emphasis on women empowerment, such as improving their access to and control over resources through a better institutional set-up, creating opportunities for non-farm income are expected to enhance pastoralists’ resilience.
