# Erratum: Vulnerability of Southern Afar pastoralists to climate variability and change, Ethiopia

**DOI:** 10.4102/jamba.v11i1.900

**Published:** 2019-12-06

**Authors:** Muluken Fenta, Andries Jordaan, Yoseph Melka

**Affiliations:** 1 Disaster Management Training and Education Centre for Africa, University of the Free State, Bloemfontein, South Africa; 2 Wondo Genet College of Forestry and Natural Resources, Hawassa University, Shashemene, Ethiopia

In the version of this article published earlier, the date of publication reflected the incorrect year. The publication date, under the heading ‘Dates’, should have been 18 Apr. 2019 instead of 18 Apr. 2018.

This correction does not alter the study’s findings of significance or overall interpretation of the study results. The publisher sincerely apologises for any inconvenience caused.

